# Genetic diversity and population structure of four Chinese rabbit breeds

**DOI:** 10.1371/journal.pone.0222503

**Published:** 2019-09-16

**Authors:** Anyong Ren, Kun Du, Xianbo Jia, Rui Yang, Jie Wang, Shi-Yi Chen, Song-Jia Lai

**Affiliations:** 1 Farm Animal Genetic Resources Exploration and Innovation Key Laboratory of Sichuan Province, Sichuan Agricultural University, Chengdu, China; 2 Animal Breeding and Genetics Key Laboratory of Sichuan Province, Sichuan Animal Science Academy, Chengdu, China; National Cheng Kung University, TAIWAN

## Abstract

There are a few well-known indigenous breeds of Chinese rabbits in Sichuan and Fujian provinces, for which the genetic diversity and population structure have been poorly investigated. In the present study, we successfully employed the restriction-site-associated DNA sequencing (RAD-seq) approach to comprehensively discover genome-wide SNPs of 104 rabbits from four Chinese indigenous breeds: 30 Sichuan White, 34 Tianfu Black, 32 Fujian Yellow and eight Fujian Black. A total of 7,055,440 SNPs were initially obtained, from which 113,973 high-confidence SNPs (read depth ≥ 3, calling rate = 100% and biallelic SNPs) were selected to study the genetic diversity and population structure. The mean polymorphism information content (PIC) and nucleotide diversity (π) of each breed slightly varied with ranging from 0.2000 to 0.2281 and from 0.2678 to 0.2902, respectively. On the whole, Fujian Yellow rabbits showed the highest genetic diversity, which was followed by Tianfu Black and Sichuan White rabbits. The principal component analysis (PCA) revealed that the four breeds were clearly distinguishable. Our results first reveal the genetic differences among these four rabbit breeds in the Sichuan and Fujian provinces and also provide a high-confidence set of genome-wide SNPs for Chinese indigenous rabbits that could be employed for gene linkage and association analyses in the future.

## Introduction

Rabbits (*Oryctolagus cuniculus*) are one of the most recently domesticated animals with an estimated history of approximately 1,400 years [1, 2]. After the initial domestication in France, more than 200 modern breeds or populations have been recognized worldwide and all of them show a considerable phenotypic variation [3, 4]. In China, there are approximately 20 indigenous and recently imported rabbit breeds, which are widely kept for their meat, fur and wool [5]. Compared to the indigenous rabbit breeds, these imported breeds are more prevalent in the Chinese rabbit industry because of their better production performances on the important economic traits [6]. However, these indigenous breeds have superior disease resistance and environmental adaptation [7], and these characteristics make them important for the sustainable development of the rabbit industry in China. Unfortunately, the genetic diversity and population structure of Chinese indigenous rabbits have not been well studied yet especially at the genome-wide level.

During the last decades, single nucleotide polymorphisms (SNPs) have become the most popular genetic markers for studying genetic diversity and population structure in wild and domestic animals. With rapid development of high-throughput sequencing techniques, restriction site-associated DNA sequencing (RAD-seq) provides a relatively cost-effective approach to obtain tens of thousands of genome-wide SNPs [8, 9]. The RAD-seq technique first employs one or more restriction enzyme(s) to randomly digest genome sequences into short fragments that are then subjected to massively parallel DNA sequencing [10]. Overall, the RAD-seq is a very prevalent approach in studies of population genetics because it has advantages for generating the relatively equally distributed SNPs suitable to reveal genetic diversity and population structure [11–13].

The objective of the present study was to discover the genome-wide SNPs by RAD-seq approach and then investigate genetic diversity and population structure of the four Chinese rabbit breeds. In addition to providing a high-confidence set of genome-wide SNP markers that could be employed for gene linkage and association analyses, the revealed inter-breed genetic differences will help us for better establishing the conservation strategies of genetic diversity and crossbreeding systems in rabbit industry.

## Materials and methods

### Ethics statement

All experimental protocols involved in this study were approved by the Institutional Animal Care and Use Committee of the College of Animal Science and Technology, Sichuan Agricultural University, Sichuan, China (No. DKYB20081003).

### Blood sampling and DNA extraction

**Fig 1:**
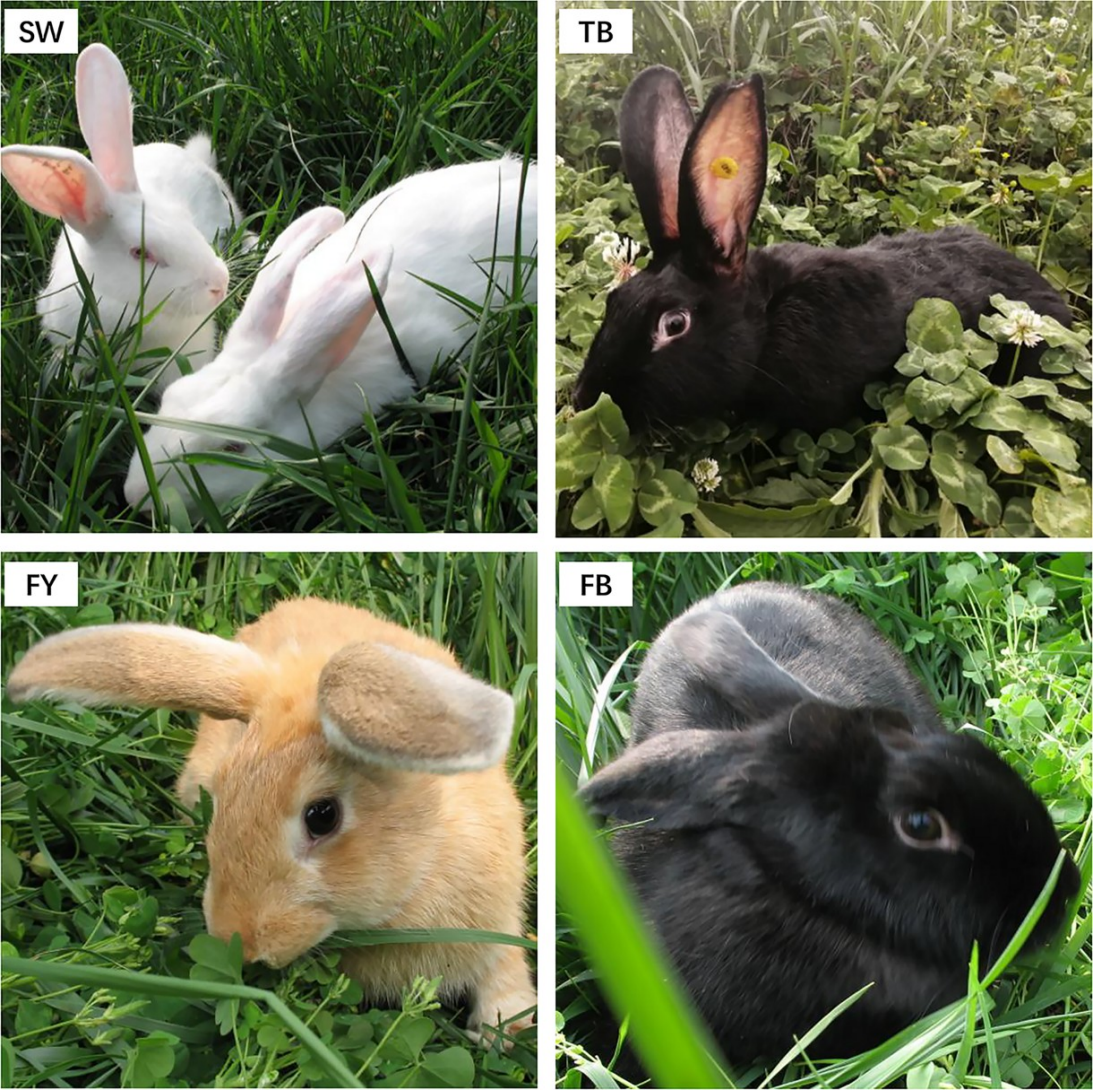
Rabbit pictures from each of the four indigenous breeds in this study.

Blood samples were randomly collected from 104 unrelated individuals from four indigenous breeds of Chinese rabbits (Fig 1), including 30 Sichuan White (SW) and 34 Tianfu Black (TB) from Sichuan province, 32 Fujian Yellow (FY) and eight Fujian Black (FB) from Fujian province. All rabbits were raised in the experimental farms of the Sichuan Agricultural University and Sichuan Animal Science Academy, and none of them had genetic relationships during the previous three generations. Total genomic DNA was extracted using the standard procedure of the Animal Genomic DNA Kit (Tiangen, Beijing), and individual DNA quality was evaluated by NanoVue Plus (GE, USA).

### Library construction and Illumina sequencing

RAD-seq sequencing libraries were constructed according to the recommended pipeline [10]. Briefly, genomic DNA (~1 μg per sample) was first digested with *EcoRI* (NEB, Beijing), onto which the P1 adaptor was ligated. Subsequently, the samples were pooled, randomly sheared, and size-selected in sequential steps. After the second adapter (P2) was added, the DNA fragments of 300 to 500 bp in length were used to construct the sequencing libraries. Finally, the Illumina HiSeq2000 platform was employed to sequence the constructed libraries and generate 150 bp paired-end reads (BioMarker Co.Ltd., Beijing).

### Quality control, read mapping and SNP calling

All the raw sequencing reads were first subjected to quality control by removing these low-quality reads, which were defined by any of three criteria: (i) reads containing low-quality bases (Q_phred_ value < 5) more than 50% of the total length, (ii) reads containing adaptor sequences, and (iii) reads containing ambiguous bases more than 10% of its total length. This filtering step of reads was performed using the fastp tool (v0.19.5) [14], after which we obtained the clean reads that were subjected to SNP calling.

All reads were mapped against the reference rabbit genome (OryCun2.0) using the BWA-MEM algorithm in BWA software (v0.7.17) [15] with default parameters. The generated SAM (Sequence Alignment/Map) files were manipulated with Picard tools (v1.134, http://broadinstitute.github.io/picard/), including the coordinate sorting and duplicate removing. Subsequently, the GATK software (v3.7) [16] was applied to SNP calling and individual genotyping according to recommendations of GATK Best Practices [17, 18]. Additionally, the local realignment around indels was conducted using GATK realignment algorithm. We further performed the hard filtering with expression of “QD < 2.0 || FS > 60.0 || MQ < 40.0 || MQRankSum < -12.5 || ReadPosRankSum < -8.0” for producing the clean SNPs. Finally, the high-confidence SNPs were finally retained for further analysis based on three criteria, including (i) coverage depth of reads ≥ 3 for every sample, (ii) calling rate of 100% (i.e., no any missing in the samples), and (iii) biallelic SNPs.

### Data analyses

First, we investigated the overall read depth and chromosomal distribution for all SNPs using the VCFtools [19]. The nucleotide diversity (π), expected heterozygosity (*H*_e_), observed heterozygosity (*H*_o_), private allele number (*A*_p_), frequency of the most frequent allele (*P*), fixation index (*F*_ST_), and inbreeding coefficient (*F*_IS_) for each breeds were computed using the ‘population’ program in Stacks (v2.2) [20]. The PopSc toolkit [21] was utilized to calculate the polymorphism information content (PIC) for each breeds. *F*_ST_ and *F*_IS_ values were computed to analyze pairwise population differences among the four breeds. To evaluate the genetic relationship among all four breeds, a principal component analysis (PCA) was conducted with GCTA (v1.26.0) [22] after converting the SNP data into PED format by PLINK (v1.07) [23]. All the related results were plotted using ggplot2 (v3.1.0) [24] from R package.

## Results

**Fig 2:**
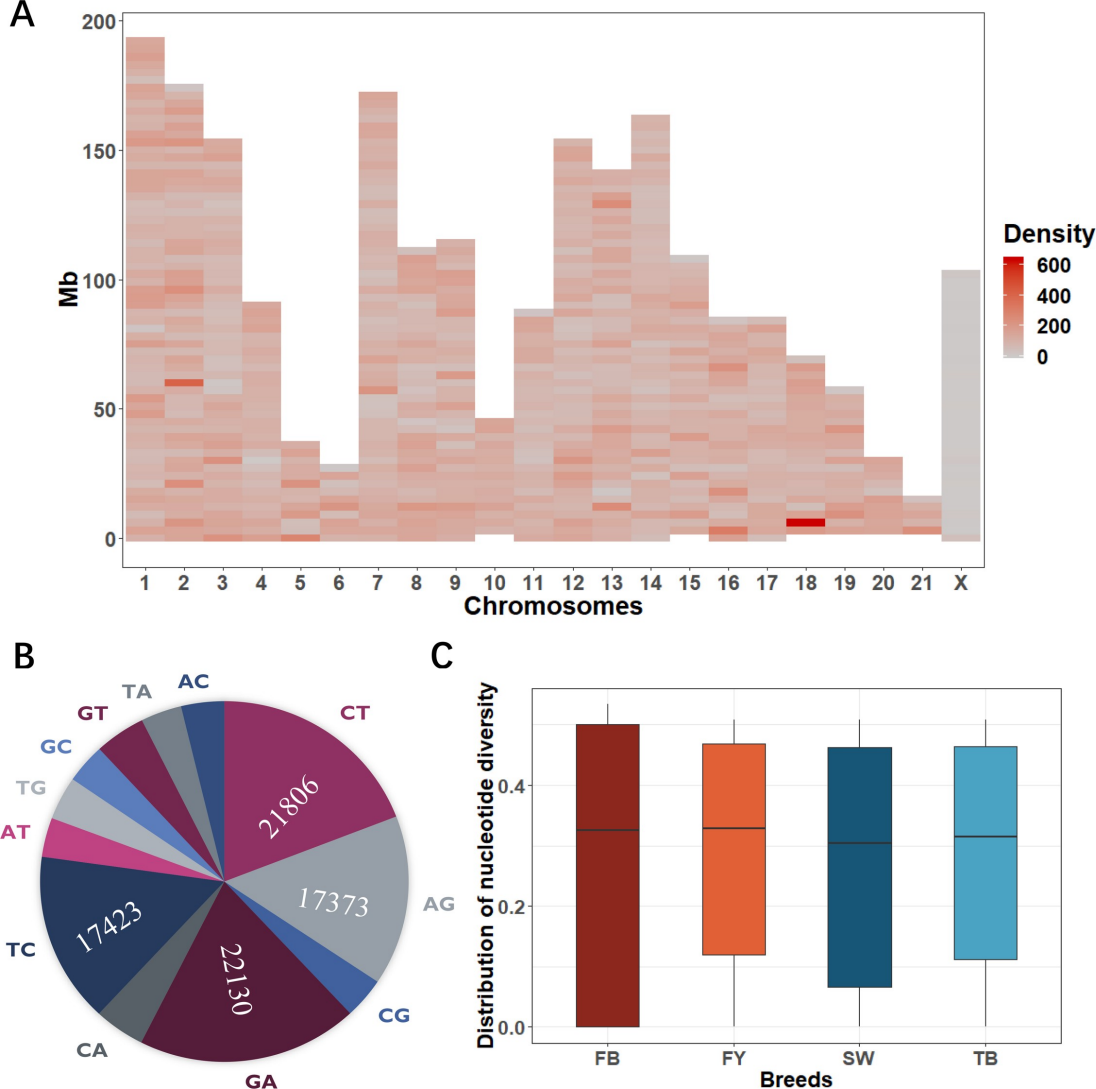
SNP distribution and nucleotide diversity. (A) Distribution density of SNPs across 22 chromosomes, in which each block represents a split bin. (B) Substitution types of SNPs in all populations. (C) Distribution density of the nucleotide diversity in four rabbit breeds.

We obtained 295 Gb of raw paired-end reads with an average of 2.83 Gb per sample, which ultimately produced 260 Gb of clean paired-end reads after the quality filtering (S1 Table). An average of 99.21% of clean reads were successfully mapped to the reference genome, by which we identified 7,955,814 raw SNPs and 7,055,440 clean SNPs, respectively. To avoid potential biases, we strictly selected a high-confidence set of 113,973 SNPs for the further analysis, among them 37,343 SNPs were located within these unplaced scaffolds. After splitting the 22 chromosomes into 750 bins of 3 Mb in size, there was an average of 102 SNPs per bin (Fig 2A). For all SNPs, we estimated an transition vs. transversion ratio of 2.23, including 78,732 transitions and 35,241 transversions (Fig 2B).

We subsequently computed six indexes in relation to the intra-breed genetic diversity for every one of the four rabbit breeds (Table 1). There were 3,679 private alleles for FY, 1,089 for FB, 1,833 for SW and 4,506 for TB, respectively. The mean frequency of the most frequent allele ranged from 0.7833 (FY) to 0.8071 (FB), the nucleotide diversity from 0.2678 (FB) to 0.2902 (FY), and the polymorphism information content from 0.2000 (FB) to 0.2281 (FY). The FB breed had the lowest expected heterozygosity, whereas the highest observed heterozygosity was observed in FY breed. We further investigated the intra-breed overall distribution of nucleotide diversity for all SNPs (Fig 2C), which showed the FB breed had the highest variation.

**Table 1:** Values of genetic diversity in four rabbit breeds using SNP data.

Breeds	*A*_p_	P	π	PIC	*H*_e_	*H*_o_
FY	3,679	0.7833	0.2902	0.2281	0.2857	0.3418
FB	1,089	0.8071	0.2678	0.2000	0.2511	0.3072
SW	1,833	0.7989	0.2689	0.2109	0.2644	0.3158
TB	4,506	0.7867	0.2871	0.2264	0.2829	0.3115

*A*_P_, private allele number; P, frequency of the most frequent allele; π, nucleotide diversity; PIC, polymorphism information content; *H*_e_, expected heterozygosity; *H*_o_, observed heterozygosity.

**Fig 3:**
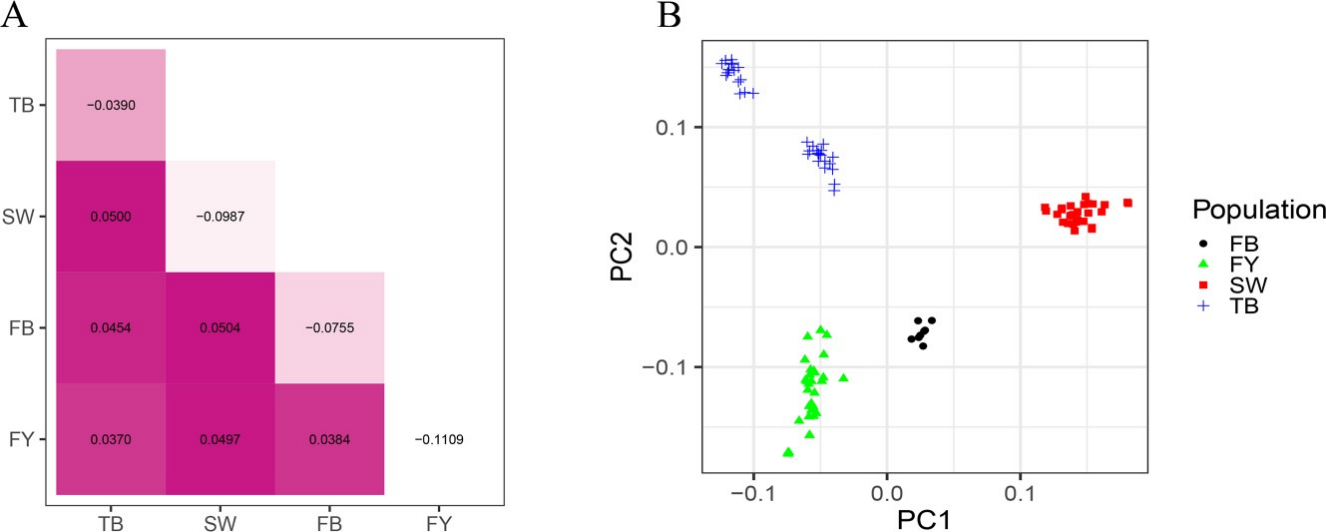
Population structure of the four rabbit breeds. (A) The plot shows the pairwise Wright’s *F*_ST_ values in the lower triangular area and *F*_IS_ values in diagonal cells. (B) Plot of pairwise principal components (PC1 and PC2) of four rabbit breeds based on the SNP data.

The pairwise comparisons of Wright’s *F*_ST_ values showed low to moderate levels of genetic differentiation among the four rabbit breeds (Fig 3A). Among them, the lowest and highest inter-breed differences were observed between FY and TB (*F*_ST_ = 0.0370) and between FB and SW (*F*_ST_ = 0.0504), respectively. The intra-population inbreeding coefficient of *F*_IS_ ranged from -0.1109 (FY) to -0.0390 (TB). Furthermore, the PCA-based clustering first revealed that all the four breeds were clearly distinguishable (Fig 3B). In addition, the individuals from FY, FB and SW breeds were clustered together with each of these breeds. In contrast, the 34 Tianfu black rabbits (TB) were divided into two distinct subgroups.

## Discussion

China has the largest volumes of consumption and production for rabbit meat, both comprising more than 60% of the world's totals [25]. Therefore, sustainable development of the Chinese rabbit industry significantly depends on a sufficient amount of genetic resources available, especially for these indigenous breeds. Although the genetic diversity and population structure of Chinese indigenous rabbits has been studied in a few sporadic reports on the basis of microsatellite markers [26, 27] and mitochondrial DNA [5], a genome-wide systematic investigation still remains to be addressed. In China, Sichuan and Fujian are the representative provinces of rabbit raising with a long history, both of them also have the well-known indigenous breeds, such as Sichuan White and Fujian Yellow rabbits. In the present study, we first discover the genome-wide SNPs comprehensively and then analyze genetic diversity and population structure of the four widely used indigenous rabbit breeds in Sichuan and Fujian provinces, which is expected to significantly facilitate the effective conservation and exploration of these genetic resources. Further, we anticipate that the SNP markers identified in the present study will be a valuable resource for conducting gene linkage and association analyses in other rabbit populations.

Our results revealed that Fujian Yellow and Fujian Black rabbits have the highest and lowest genetic diversity, respectively; whereas only small differences of genetic diversity were observed among the four studied breeds on the whole. In addition, we should be cautious for the conclusion that Fujian Black rabbits have the lowest genetic diversity because only eight individuals were sampled in the present study. Based on 30 microsatellite markers, Xie and colleagues [26] previously reported that the polymorphism information content and expected heterozygosity of Fujian Yellow rabbits were 0.6766 and 0.7324, both of which are substantially higher than the corresponding values computed in the present study. Unfortunately, we are unable to compare the four breeds of Chinese indigenous rabbits with other Chinese rabbit breeds or with widely used European rabbit breeds because the allele frequency data of reference populations were unavailable. Interestingly, we also observed that the four Chinese rabbit breeds in the present study could be fully separated from each other based on the PCA-based clustering, which indicates that there were significant genetic differences among these populations.

In conclusion, we comprehensively discover the genome-wide SNPs and systematically investigate the genetic diversity and population structure for four Chinese rabbit breeds. The results will help us to better conserve and explore these genetic resources, and also facilitate the future studies of gene linkage and association analyses in these and other rabbit populations.

## Supporting information

S1 Table Sequencing and quality filtering of reads. (DOCX)
