# Supplementary material for: Genetic diversity and population structure of four Chinese rabbit breeds
Source: PLoS One. 2019 Sep 16;14(9):e0222503. doi: 10.1371/journal.pone.0222503 (PMC6746397; doi:10.1371/journal.pone.0222503)
Supplement: S1 Table — (DOCX) [file pone.0222503.s001.docx]

**S1 Table.** Sequencing results and quality filtering of reads

| **SampleID** | **Raw reads(M)** | **Clean reads(M)** | **Raw base(G)** | **Clean base(G)** | **Q20(%)** | **Q30(%)** | **GC content(%)** |
| --- | --- | --- | --- | --- | --- | --- | --- |
| **D17103_1** | 20.44 | 18.12 | 3.004 | 2.663 | 96.08 | 90.40 | 41.45 |
| **D17103_2** | 20.57 | 18.04 | 3.034 | 2.661 | 96.20 | 90.41 | 41.71 |
| **D17203** | 18.81 | 16.71 | 2.783 | 2.473 | 96.13 | 90.51 | 41.48 |
| **D27106** | 19.93 | 17.69 | 2.950 | 2.619 | 96.31 | 90.86 | 41.56 |
| **D27202** | 17.59 | 15.41 | 2.586 | 2.265 | 96.20 | 90.64 | 41.56 |
| **D27206** | 19.04 | 16.85 | 2.779 | 2.459 | 96.27 | 90.65 | 41.38 |
| **D36605** | 13.69 | 12.04 | 1.998 | 1.757 | 96.16 | 90.43 | 41.52 |
| **D36V05** | 15.83 | 14.10 | 2.326 | 2.072 | 96.27 | 90.76 | 41.57 |
| **D37201** | 15.92 | 14.19 | 2.341 | 2.087 | 96.08 | 90.41 | 41.46 |
| **D46Y02** | 21.17 | 18.75 | 3.101 | 2.747 | 96.28 | 90.80 | 41.55 |
| **D47105** | 18.52 | 16.17 | 2.731 | 2.386 | 96.02 | 90.16 | 41.89 |
| **D47202** | 21.80 | 19.01 | 3.204 | 2.795 | 96.19 | 90.61 | 41.58 |
| **D56V05** | 19.48 | 17.27 | 2.873 | 2.547 | 96.12 | 90.48 | 41.45 |
| **D56Y01** | 22.19 | 19.34 | 3.251 | 2.833 | 96.03 | 90.19 | 41.76 |
| **D57102** | 19.78 | 17.23 | 2.887 | 2.515 | 96.25 | 90.71 | 41.69 |
| **D57203** | 17.77 | 15.62 | 2.603 | 2.288 | 96.19 | 90.39 | 41.50 |
| **D66Y05** | 17.60 | 15.42 | 2.570 | 2.251 | 96.22 | 90.43 | 41.60 |
| **D67101** | 22.74 | 20.12 | 3.354 | 2.968 | 96.30 | 90.85 | 41.56 |
| **D67103** | 13.46 | 11.94 | 1.965 | 1.744 | 96.06 | 90.01 | 42.02 |
| **D67105** | 21.67 | 19.19 | 3.196 | 2.830 | 96.28 | 90.81 | 41.55 |
| **D77103** | 17.35 | 15.19 | 2.568 | 2.248 | 96.22 | 90.68 | 41.54 |
| **D77104** | 19.60 | 17.11 | 2.890 | 2.524 | 96.21 | 90.65 | 41.54 |
| **D77205** | 18.05 | 16.04 | 2.645 | 2.351 | 96.08 | 90.40 | 41.47 |
| **D87201** | 13.13 | 11.73 | 1.917 | 1.713 | 96.03 | 90.15 | 41.51 |
| **D87205** | 22.15 | 19.30 | 3.267 | 2.846 | 96.23 | 90.70 | 41.52 |
| **D96901** | 21.34 | 18.81 | 3.111 | 2.746 | 96.33 | 90.87 | 41.68 |
| **DG7205** | 20.88 | 18.22 | 3.059 | 2.670 | 96.22 | 90.68 | 41.48 |
| **DP7103** | 15.34 | 13.67 | 2.239 | 1.995 | 96.30 | 90.72 | 41.62 |
| **DP7202** | 20.55 | 18.23 | 3.032 | 2.688 | 96.12 | 90.49 | 41.48 |
| **EG6704** | 22.57 | 19.98 | 3.318 | 2.937 | 96.26 | 90.77 | 41.54 |
| **Famale2827** | 21.59 | 19.17 | 3.174 | 2.818 | 96.23 | 90.60 | 41.36 |
| **Famale2898** | 18.21 | 16.22 | 2.695 | 2.400 | 96.31 | 90.77 | 41.44 |
| **Famale2958** | 17.41 | 15.52 | 2.559 | 2.281 | 96.22 | 90.58 | 41.35 |
| **Famale7322** | 19.15 | 17.03 | 2.825 | 2.512 | 96.24 | 90.62 | 41.47 |
| **Famale7461** | 21.09 | 18.73 | 3.111 | 2.762 | 96.26 | 90.67 | 41.36 |
| **Male2001** | 19.66 | 17.44 | 2.881 | 2.555 | 96.25 | 90.63 | 41.31 |
| **Male2322** | 14.44 | 12.93 | 2.109 | 1.888 | 96.18 | 90.38 | 41.57 |
| **1** | 11.31 | 9.97 | 1.652 | 1.456 | 95.98 | 89.97 | 40.89 |
| **106** | 18.28 | 16.06 | 2.696 | 2.369 | 96.20 | 90.42 | 41.28 |
| **118** | 12.09 | 10.90 | 1.765 | 1.591 | 96.23 | 90.31 | 41.48 |
| **128** | 16.21 | 13.88 | 2.383 | 2.041 | 96.18 | 90.52 | 40.98 |
| **150** | 15.17 | 13.65 | 2.222 | 1.999 | 96.19 | 90.36 | 41.46 |
| **151** | 13.88 | 12.47 | 2.027 | 1.821 | 96.20 | 90.34 | 41.63 |
| **152** | 11.75 | 10.39 | 1.739 | 1.538 | 96.20 | 90.42 | 41.53 |
| **156** | 18.44 | 16.17 | 2.710 | 2.377 | 96.20 | 90.43 | 41.30 |
| **162** | 21.31 | 18.21 | 3.122 | 2.667 | 96.16 | 90.45 | 41.28 |
| **165** | 16.87 | 15.15 | 2.488 | 2.235 | 96.20 | 90.38 | 41.56 |
| **166** | 20.71 | 18.19 | 3.045 | 2.674 | 96.17 | 90.36 | 41.36 |
| **167** | 15.26 | 13.18 | 2.229 | 1.924 | 96.03 | 90.06 | 41.40 |
| **174** | 19.51 | 16.70 | 2.887 | 2.472 | 96.18 | 90.52 | 41.13 |
| **175** | 19.01 | 16.27 | 2.805 | 2.400 | 96.16 | 90.46 | 41.51 |
| **176** | 17.67 | 15.08 | 2.579 | 2.202 | 96.18 | 90.47 | 41.35 |
| **177** | 22.07 | 18.84 | 3.255 | 2.779 | 96.19 | 90.53 | 41.19 |
| **180** | 14.76 | 12.70 | 2.170 | 1.867 | 96.16 | 90.46 | 41.19 |
| **18210** | 18.24 | 16.06 | 2.682 | 2.360 | 96.02 | 90.13 | 41.22 |
| **2** | 27.41 | 23.76 | 4.002 | 3.469 | 96.09 | 90.30 | 41.63 |
| **2614** | 19.02 | 16.70 | 2.795 | 2.455 | 96.00 | 90.10 | 41.33 |
| **3** | 19.02 | 17.08 | 2.796 | 2.511 | 96.20 | 90.37 | 41.49 |
| **4** | 14.53 | 13.10 | 2.136 | 1.925 | 96.17 | 90.32 | 41.39 |
| **5** | 18.40 | 16.54 | 2.724 | 2.448 | 96.19 | 90.35 | 41.36 |
| **6** | 15.15 | 13.64 | 2.235 | 2.013 | 96.16 | 90.29 | 41.51 |
| **7331** | 16.82 | 14.94 | 2.456 | 2.181 | 95.74 | 89.32 | 40.99 |
| **9062** | 18.55 | 16.32 | 2.736 | 2.407 | 96.00 | 90.10 | 41.50 |
| **9200** | 19.54 | 17.16 | 2.8892 | 2.539 | 96.06 | 90.22 | 41.24 |
| **F0111** | 24.35 | 21.48 | 3.567 | 3.147 | 95.95 | 89.89 | 41.40 |
| **F0513** | 21.94 | 19.17 | 3.237 | 2.827 | 96.42 | 91.14 | 41.32 |
| **F0518** | 18.91 | 17.33 | 2.770 | 2.539 | 95.92 | 89.78 | 41.05 |
| **F0761** | 18.63 | 17.07 | 2.738 | 2.509 | 95.89 | 89.71 | 41.08 |
| **F0764** | 27.54 | 24.24 | 4.048 | 3.563 | 95.91 | 89.78 | 41.34 |
| **F0826** | 16.91 | 14.86 | 2.486 | 2.184 | 96.41 | 91.10 | 41.42 |
| **F0885** | 16.68 | 14.78 | 2.461 | 2.179 | 95.82 | 89.62 | 40.92 |
| **F0890** | 23.07 | 20.08 | 3.369 | 2.931 | 96.46 | 91.20 | 41.53 |
| **F0892** | 20.23 | 17.89 | 2.954 | 2.612 | 96.16 | 90.54 | 41.73 |
| **F0913** | 17.01 | 14.95 | 2.483 | 2.182 | 96.41 | 91.01 | 41.55 |
| **F0921** | 23.87 | 20.83 | 3.521 | 3.072 | 96.45 | 91.20 | 41.37 |
| **F0936** | 20.86 | 18.47 | 3.066 | 2.715 | 95.90 | 89.77 | 41.36 |
| **F0965** | 22.22 | 19.43 | 3.256 | 2.846 | 96.42 | 91.13 | 41.49 |
| **F0968** | 19.11 | 16.92 | 2.828 | 2.504 | 95.81 | 89.59 | 40.96 |
| **F0970** | 19.10 | 17.50 | 2.817 | 2.581 | 95.97 | 89.88 | 40.94 |
| **F0971** | 14.82 | 13.61 | 2.193 | 2.015 | 95.95 | 89.83 | 41.27 |
| **F1014** | 18.18 | 16.13 | 2.672 | 2.372 | 95.74 | 89.45 | 41.06 |
| **F1026** | 21.42 | 18.94 | 3.127 | 2.765 | 95.86 | 89.55 | 41.59 |
| **F1034** | 23.55 | 20.57 | 3.486 | 3.044 | 96.45 | 91.20 | 41.73 |
| **F1035** | 20.33 | 17.98 | 2.978 | 2.634 | 95.79 | 89.56 | 40.89 |
| **F1040** | 23.67 | 20.81 | 3.457 | 3.038 | 96.01 | 89.99 | 41.38 |
| **F1045** | 22.50 | 19.65 | 3.307 | 2.888 | 96.41 | 91.10 | 41.56 |
| **F1050** | 24.96 | 21.89 | 3.644 | 3.197 | 95.84 | 89.64 | 41.19 |
| **F1055** | 22.17 | 19.58 | 3.271 | 2.889 | 95.81 | 89.60 | 40.92 |
| **F1067** | 26.80 | 23.60 | 3.953 | 3.481 | 95.95 | 89.88 | 41.36 |
| **F1071** | 21.79 | 19.24 | 3.203 | 2.829 | 95.76 | 89.49 | 40.98 |
| **F1075** | 19.53 | 17.84 | 2.851 | 2.605 | 95.98 | 89.89 | 41.09 |
| **F1076** | 24.90 | 21.97 | 3.685 | 3.252 | 95.96 | 89.89 | 41.44 |
| **F1081** | 16.65 | 15.27 | 2.456 | 2.252 | 95.95 | 89.83 | 40.99 |
| **F1092** | 23.15 | 20.43 | 3.414 | 3.014 | 95.93 | 89.84 | 41.38 |
| **F1095** | 13.93 | 12.83 | 2.048 | 1.885 | 95.89 | 89.70 | 41.19 |
| **F1100** | 13.22 | 12.16 | 1.931 | 1.776 | 95.89 | 89.60 | 41.04 |
| **3156** | 21.62 | 18.95 | 3.168 | 2.776 | 96.05 | 90.21 | 40.97 |
| **3238** | 16.18 | 14.29 | 2.363 | 2.086 | 95.92 | 89.82 | 41.09 |
| **3245** | 22.79 | 19.86 | 3.373 | 2.940 | 96.08 | 90.31 | 41.40 |
| **3314** | 21.42 | 18.71 | 3.149 | 2.750 | 96.06 | 90.26 | 41.44 |
| **4211** | 20.08 | 17.53 | 2.932 | 2.559 | 96.04 | 90.16 | 41.15 |
| **4262** | 23.45 | 20.40 | 3.447 | 2.999 | 96.07 | 90.29 | 41.42 |
| **4334** | 25.00 | 21.69 | 3.687 | 3.199 | 96.09 | 90.34 | 41.14 |
| **4362** | 21.89 | 19.16 | 3.229 | 2.826 | 96.05 | 90.21 | 41.09 |
| **Average** | 19.29 | 17.02 | 2.834 | 2.501 | 96.12 | 90.32 | 41.38 |
| **Total** | 2005.92 | 1769.73 | 294.79 | 260.08 | - | - | - |
